# Supplementary figures and images for: Diagnostic accuracy of direct agglutination test, rK39 ELISA and six rapid diagnostic tests among visceral leishmaniasis patients with and without HIV coinfection in Ethiopia
Source: PLoS Negl Trop Dis. 2020 Dec 31;14(12):e0008963. doi: 10.1371/journal.pntd.0008963 (PMC7774845; doi:10.1371/journal.pntd.0008963)

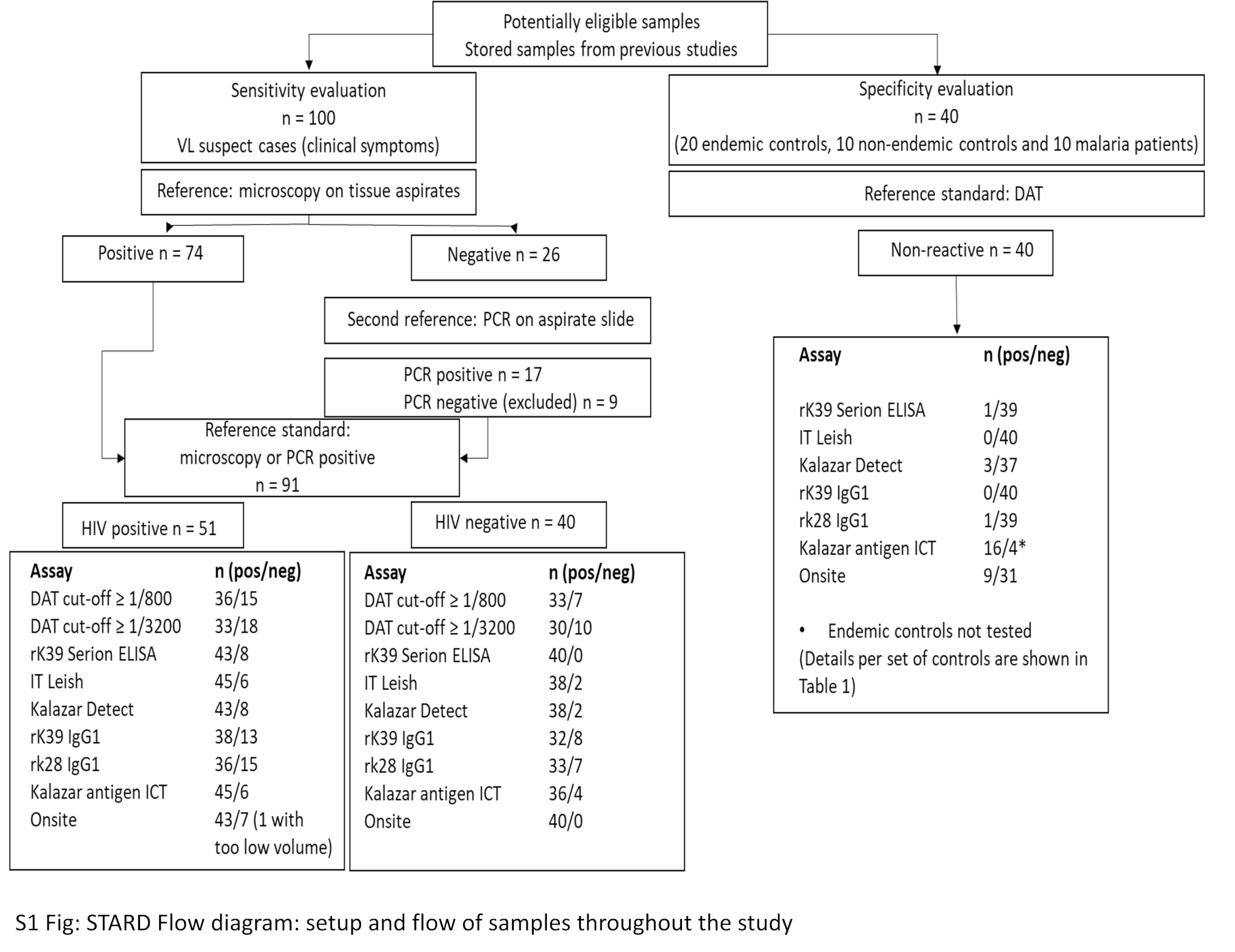

Supplement: S1 Fig — (TIF) [file pntd.0008963.s001.tif]
