# Supplementary material for: Diagnostic accuracy of direct agglutination test, rK39 ELISA and six rapid diagnostic tests among visceral leishmaniasis patients with and without HIV coinfection in Ethiopia
Source: PLoS Negl Trop Dis. 2020 Dec 31;14(12):e0008963. doi: 10.1371/journal.pntd.0008963 (PMC7774845; doi:10.1371/journal.pntd.0008963)
Supplement: Attachment — Submitted filename: PNTD-D-20-01173_reviewer.pdf [file pntd.0008963.s003.pdf]

PLOS Neglected Tropical Diseases

Diagnostic accuracy of direct agglutination test, rK39 ELISA and six rapid diagnostic tests among visceral leishmaniasis patients with and without HIV coinfection in Ethiopia

--Manuscript Draft--

**Manuscript Number:**	PNTD-D-20-01173
**Full Title:**	Diagnostic accuracy of direct agglutination test, rK39 ELISA and six rapid diagnostic tests among visceral leishmaniasis patients with and without HIV coinfection in Ethiopia
**Short Title:**	Diagnostic accuracy of VL antibody and antigen tests in HIV coinfected patients
**Article Type:**	Research Article
**Keywords:**	Leishmania; HIV; rapid diagnostic tests; DAT; ELISA; method comparison
**Abstract:**	Background<br>Diagnosis of de novo visceral leishmaniasis (VL) infection in Ethiopia is established by use of a rapid diagnostic test (RDT) detecting antibodies against rK39, direct agglutination test (DAT) and microscopy according to the national algorithm. The performance of individual tests and algorithm is variable and depends on several factors, one being HIV status. Limited data are available on the performance of tests in VL-HIV coinfected patients. We aimed to assess performance difference between VL and VL-HIV coinfected Ethiopian patients.<br>Methods/ Results<br>Assessment of the performance of DAT, rK39 ELISA and six RDT for the diagnosis of VL was done on a panel of 91 stored serum and plasma samples of 'de novo' suspected VL patients, with HIV coinfection (n=51) and without (n=40). A combined reference standard was used: either positive microscopy on tissue aspirates, or in case of negative microscopy, positive PCR results on the aspirate slide. Additionally, endemic healthy controls (n=20), non-endemic controls (n=10) and patients with confirmed malaria infection (n=10) were tested for specificity evaluation.<br>Sensitivities ranged from 69.2% for DAT (applied cut-off ≥ 1/3200) to 92.2% for the Onsite RDT, whereas specificities ranged from 20.0% for Kalazar ICT to 100% for IT Leish and rK39 IgG1. Sensitivities from all assays decreased upon stratification according to HIV status but was only significantly different for rK39 Serion ELISA (p-value 0.0084) and the Onsite RDT (p-value 0.0159).<br>Conclusions<br>Performance of commercially available assays for VL on samples from Northern-Ethiopian patients varied widely with a substantial decrease in sensitivity in the VL-HIV coinfected group. Clear guidelines on minimal performance criteria of individual tests and algorithms, but also against which reference these should be compared are needed.
**Additional Information:**	
**Question**	**Response**
**Financial Disclosure**<br><br>Enter a financial disclosure statement that describes the sources of funding for the work included in this submission. Review the submission guidelines for detailed requirements. View published research articles from *PLOS NTDs* for specific examples.<br><br>This statement is required for submission and **will appear in the published article** if the submission is accepted. Please make	This study was part of a larger project supported by the Department of Economy, Science and Innovation of the Flemish Government, and the Belgian Directorate General for Development Cooperation under the ITM-DGDC framework agreement FA-III & FAIV.<br>B.C.B.H. work was supported by funding from the European Union's Horizon 2020 research and innovation programme under the Marie Sklodowska-Curie actions (grant agreement No 642609).<br>The funders had no role in study design, data collection and analysis, decision to publish, or preparation of the manuscript.

sure it is accurate.<br><br>**Unfunded studies**<br><br>Enter: *The author(s) received no specific funding for this work.*<br><br>**Funded studies**<br><br>Enter a statement with the following details:<br>• Initials of the authors who received each award<br>• Grant numbers awarded to each author<br>• The full name of each funder<br>• URL of each funder website<br>• Did the sponsors or funders play any role in the study design, data collection and analysis, decision to publish, or preparation of the manuscript?<br>• **NO** - Include this sentence at the end of your statement: *The funders had no role in study design, data collection and analysis, decision to publish, or preparation of the manuscript.*<br>• **YES** - Specify the role(s) played.<br><br>* typeset	
**Competing Interests**<br><br>Use the instructions below to enter a competing interest statement for this submission. On behalf of all authors, disclose any competing interests that could be perceived to bias this work—acknowledging all financial support and any other relevant financial or non-financial competing interests.<br><br>This statement **will appear in the published article** if the submission is accepted. Please make sure it is accurate. View published research articles from *PLOS NTDs* for specific examples.	The authors have declared that no competing interest exist.

**NO authors have competing interests**<br><br>Enter: *The authors have declared that no competing interests exist.*<br><br>**Authors with competing interests**<br><br>Enter competing interest details beginning with this statement:<br><br>*I have read the journal's policy and the authors of this manuscript have the following competing interests: [insert competing interests here]*<br><br>* typeset	
**Data Availability**<br><br>Authors are required to make all data underlying the findings described fully available, without restriction, and from the time of publication. PLOS allows rare exceptions to address legal and ethical concerns. See the PLOS Data Policy and FAQ for detailed information.<br><br>A Data Availability Statement describing where the data can be found is required at submission. Your answers to this question constitute the Data Availability Statement and **will be published in the article**, if accepted.	No - some restrictions will apply

**Important:** Stating 'data available on request from the author' is not sufficient. If your data are only available upon request, select 'No' for the first question and explain your exceptional situation in the text box.<br><br>Do the authors confirm that all data underlying the findings described in their manuscript are fully available without restriction?	
**Describe where the data may be found in full sentences. If you are copying our sample text, replace any instances of XXX with the appropriate details.**<br><br>• If the data are **held or will be held in a public repository**, include URLs, accession numbers or DOIs. If this information will only be available after acceptance, indicate this by ticking the box below. For example: *All XXX files are available from the XXX database (accession number(s) XXX, XXX.).*<br>• If the data are all contained **within the manuscript and/or Supporting Information files**, enter the following: *All relevant data are within the manuscript and its Supporting Information files.*<br>• If neither of these applies but you are able to provide **details of access elsewhere**, with or without limitations, please do so. For example:<br><br>*Data cannot be shared publicly because of [XXX]. Data are available from the XXX Institutional Data Access / Ethics Committee (contact via XXX) for researchers who meet the criteria for access to confidential data.*<br><br>*The data underlying the results presented in the study are available*	Data cannot be shared publicly because of patient confidentiality. Data are available from the Institute of Tropical Medicine Institutional Data Access Committee (contact via ITMresearchdataaccess@itg.be) for researchers who meet the criteria for access to confidential data.

from (include the name of the third party and contact information or URL).<br>• This text is appropriate if the data are owned by a third party and authors do not have permission to share the data.<br><br>* typeset	
Additional data availability information:	

Manuscript

Click here to access/download;Manuscript;Manuscript 2020629.doc

Diagnostic accuracy of direct agglutination test, rK39 ELISA and six rapid diagnostic tests among visceral leishmaniasis patients with and without HIV coinfection in Ethiopia

Short title: Diagnostic accuracy of VL antibody and antigen tests in HIV coinfected patients

M. Kassa[1], S. Abdellati[2], L. Cnops[2,] ,B.C. Bremer Hinckel[3], Arega Yeshanew[1], Wasihun Hailemichael[4], F. Vogt[2], W. Adriaensen[2], P. Mertens[3], E. Diro[1], J. van Griensven[2], D. Van den Bossche[2]

[1] Leishmaniasis Research and Treatment Centre, University of Gondar, Gondar, Ethiopia

[2] Department of Clinical Sciences, Institute of Tropical Medicine, Antwerp, Belgium

[3] Coris BioConcept, Gembloux

[4] Department of Immunology and Molecular Biology, Biomedical Sciences, University of Gondar, Ethiopia

Corresponding author: dvandenbossche@itg.be (DVDB)

Abstract (word count: 277)

Background

Diagnosis of de novo visceral leishmaniasis (VL) infection in Ethiopia is established by use of a rapid diagnostic test (RDT) detecting antibodies against rK39, direct agglutination test (DAT) and microscopy according to the national algorithm. The performance of individual tests and algorithm is variable and depends on several factors, one being HIV status. Limited data are available on the performance of tests in VL-HIV coinfected patients. We aimed to assess performance difference between VL and VL-HIV coinfected Ethiopian patients.

Methods/ Results

Assessment of the performance of DAT, rK39 ELISA and six RDT for the diagnosis of VL was done on a panel of 91 stored serum and plasma samples of 'de novo' suspected VL patients, with HIV coinfection (n=51) and without (n=40). A combined reference standard was used: either positive microscopy on tissue aspirates, or in case of negative microscopy, positive PCR results on the aspirate slide. Additionally, endemic healthy controls (n=20), non-endemic controls (n=10) and patients with confirmed malaria infection (n=10) were tested for specificity evaluation.

Sensitivities ranged from 69.2% for DAT (applied cut-off ≥ 1/3200) to 92.2% for the Onsite RDT, whereas specificities ranged from 20.0% for Kalazar ICT to 100% for IT Leish and rK39 IgG1. Sensitivities from all assays decreased upon stratification according to HIV status but was only significantly different for rK39 Serion ELISA (p-value 0.0084) and the Onsite RDT (p-value 0.0159).

Conclusions

Performance of commercially available assays for VL on samples from Northern-Ethiopian patients varied widely with a substantial decrease in sensitivity in the VL-HIV coinfected group. Clear guidelines on minimal performance criteria of individual tests and algorithms, but also against which reference these should be compared are needed.

Author summary (word count: 181)

In northwest Ethiopia the rates of VL patients coinfected with HIV ranges from 20 to 40%. Limited data are available on the sensitivity and specificity of antibody and antigen detection tests for VL diagnosis in HIV coinfected. Often tests are implemented without prior verification in its specific setting. However, many variables such as regional differences, molecular divergency, age of affected populations and HIV status influence performance and may substantially impact testing algorithms and outcomes.

Several tests were evaluated on a panel of samples obtained in a highly endemic Ethiopian region. rK28 antigen based rapid tests were included as they have previously shown higher sensitivity compared to rk39 based antigen tests. Though this was not confirmed here. Performance was also compared between HIV coinfected and non-HIV infected VL patients. Not all tests equally declined in sensitivity in the HIV coinfected group. Clear guidelines on minimal sensitivity and specificity, with differentiation between HIV and non-HIV infected patients are needed. Even when a national algorithm is available, verification of performance in a specific setting with selected tests and proposed criteria for acceptance remains necessary.

Text (word count: 4468)

Introduction

Leishmaniasis presents as a spectrum of diseases and is caused by obligate intracellular protozoa of the genus *Leishmania* which is transmitted by sand flies of the genus *Phlebotomus*. The three main clinical forms of the disease are the cutaneous (CL) form, mucocutaneous (MCL) and the potentially fatal visceral form, visceral leishmaniasis (VL), also called kala-azar [1]. Human VL is present worldwide, but over 90% of cases are found in a few highly-endemic countries (Brazil, Ethiopia, India, Kenya, Somalia, South Sudan and Sudan) [2]. VL is caused by members of the *L. donovani* complex, more specifically the closely related species *L. infantum* and *L. donovani*, the latter being responsible for kala-azar in East Africa.

Patients with kala-azar present with fever, splenomegaly, and weight loss. Clinical diagnosis of VL is inaccurate and can be difficult in endemic settings as several causes of febrile splenomegaly exist, notably malaria [3]. A direct parasitological diagnosis of VL is obtained through microscopy, culture and PCR on tissue aspirates. Obtaining tissue aspirates is invasive and well-trained health professionals are needed to perform this ~~high-risk~~ sampling. However, microscopy lacks sensitivity whereas culture entails weeks to yield a final result and is vulnerable to bacterial contamination. In recent years, molecular techniques have been shown to have the highest diagnostic sensitivity in aspirate or tissue samples, and to be highly specific. However, in resource-limited settings, the use of the PCR remains largely restricted to referral hospitals and well-equipped research centers [4]. Alternative diagnosis by urinary antigen detection or antibody detection through direct agglutination test (DAT), immunofluorescence assays (IFA) and enzyme-linked immunosorbent assays (ELISA) require experienced laboratory technicians, specific laboratory equipment and is time consuming. As many VL patients are poor and live in remote areas, easy and rapid tools for the diagnosis of VL should be selected in

accordance with WHO ASSURED criteria (affordable, sensitive, specific, user-friendly, rapid, equipment free and deliverable where required) [5]. Rapid diagnostic tests (RDTs) for *Leishmania* antibody detection have been implemented in endemic settings for over a decade. In combination with clinical symptoms, RDTs provide high diagnostic accuracy in suspected first-time episodes of VL [6-7]. Performance of commercially available differs throughout regions depending on the antigen used, age of the affected population and the immune status of the patient. Experience with RDT mainly consists of RDTs with antibody detection specifically against recombinant K39 (rK39), a 39-amino acid repeat that is part of a kinesin-related protein which is conserved within the *L. donovani* complex. A limitation of the rK39 based assays is its lower sensitivity in East Africa compared to the Indian subcontinent [1,8-9]. A new synthetic polyprotein was developed, rk28 which is a fusion molecule of *L. donovani* haspb1, haspb2 and LdK39 tandem repeat regions [10]. Modest improvement in sensitivity of rK28 RDTs over rK39 RDTs was reported in East Africa [11].

VL is highly endemic in both East Africa and the Indian subcontinent but has relatively more VL-HIV coinfections in East-Africa. VL-HIV coinfection rates in Ethiopia range from 20 to 40% [12]. Few studies have assessed the performance of serological assays in the VL-HIV coinfected population in East Africa, but current data suggests a lower sensitivity in VL-HIV coinfected patients [12]. Our objective was to assess the performance of different antibody tests and one antigen test for the diagnosis of new VL cases within and between a HIV negative and HIV coinfected patients in an VL- endemic region in Northern Ethiopia.

Materials and methods

Study design

A diagnostic accuracy study on stored samples was conducted to assess the performance of different RDTs, DAT and IgG ELISA for the diagnosis of VL in HIV positive and HIV negative patients. The study was carried out at the Leishmania Research and Treatment Center (LRTC), Gondar, Ethiopia and the Clinical Reference Laboratory, Institute of Tropical Medicine, Antwerp, Belgium. The study design followed the STARD guidelines for presentation of diagnostic studies [13].

A panel of biobanked serum and plasma samples from studies performed at LRTC were used for the comparison of the different tests. A confirmed case was defined by either positive microscopy on tissue aspirates, or in case of negative microscopy, positive PCR results on the aspirate slide (reference standard). One hundred samples were selected from suspected VL patients (fever for >2 weeks, splenomegaly and/or abdominal swelling and/or weight loss) with no previous VL history and stratified by density and HIV status. Out of these, 74 (45 HIV/ 29 non-HIV) were confirmed VL positive by microscopy on spleen (n=51), bone marrow (n=22) and lymph node (n=1) aspirates. Parasite density grades were 1 (6.8%), 2 (20.3%), 3 (13.5%), 4 (16.2%) 5 (17.6%) or 6 (25.7%). For 26 patients (11 HIV/15 non-HIV) no amastigotes were detected in spleen (n=18) or bone marrow (n=8), however 17 were positive by PCR performed on the aspirate slide. Nine VL suspect cases that could not be confirmed by either microscopy or PCR on slide were excluded from the analysis. This resulted in a total of 91 VL laboratory confirmed VL cases for RDT evaluation, of which 51 came from HIV coinfected patients.

Additionally, 40 samples from non-VL patients were evaluated. Samples from twenty endemic healthy Ethiopian controls, who tested negative by direct agglutination testing (DAT), were collected and analyzed with all RDTs and rK39 ELISA, except for the Kalazar antigen ICT. Ten left-over samples from Belgian travelers presenting at the polyclinic of ITM without VL, as tested by DAT, were selected as non-endemic controls; malaria was excluded by microscopy and RDTs in these patients. Ten malaria positive were included to evaluate cross-reactivity (4 *P.*

falciparum, 3 *P. vivax*, 1 *P. ovale*, 1 *P. malariae* and 1 *P. falciparum* and *P. ovale* mixed infection). All samples were stored at -80°C.

Diagnostic tests

Microscopy was performed on tissue aspirates from either bone marrow, spleen or lymph node. Slides were examined with the 100x objective after being air dried, fixed with methanol and stained with Giemsa 1:10 solution. Parasite density is estimated based on World Health Organization recommended average amastigote density grading of slides [14], grade 1 to 6.

The Direct agglutination test (DAT, Institute of Tropical Medicine – Antwerp (ITM), Belgium) was conducted with a freeze-dried version of the DAT antigen composed of fixed, trypsin-treated and stained promastigotes of *L. donovani*. DAT testing was done as previously described [15]. For result interpretation, two different cut-offs were applied, ≥ 1/800 and ≥ 1/3200.

Leishmania IgG ELISA (Serion Leishmania IgG ELISA, Virion-Serion, Würzburg, Germany) is a quantitative immunoassay which detects IgG antibodies against *Leishmania* spp. by use of the recombinant antigen K39 (rK39) and was performed according to manufacturer's instructions. Results are expressed in U/mL (negative: <10U/mL; borderline: 10 – 15 U/mL; positive: ≥15 U/mL). Borderline results were considered positive for statistical analysis.

A *Leishmania* antigen detection test (Kalazar ICT, Xinjier Biotechnology Co, Shanghai, China) was selected for evaluation of presence of *Leishmania* antigen in serum or plasma. IT-Leish (Biorad, USA), Kalazar Detect Rapid test (InBios, USA) and OnSite Leishmania Ab Rapid test (CTK, USA) are commercially available immunochromatographic tests for the qualitative detection of anti-leishmanial antibodies based on reaction with rK39 antigen (anti-rK39) for the first two and with rK28 antigen (anti-rK28) for the latter test. Two prototype tests detecting IgG1 against either rK39 or rK28 (Coris BioConcept, Belgium) were evaluated. These consisted of a cassette with a nitrocellulose membrane sensitized with rK28 (concentration 0.25mg/mL) or

rK39 (concentration 0.6mg/mL). Anti-human IgG1 specific antibody labelled with colloidal gold was dried onto the conjugate pad. The control line consisted of goat anti-chicken (GAC) antibodies which was detected by chicken IgY conjugate, impregnated together with the anti-human IgG1 conjugate. 3.5µL of serum or plasma was dispensed on the nitrocellulose membrane, followed by 150µL of buffer in the buffer well. Results were read after 15 minutes. A test was deemed valid if a red control band developed where the control antibody was present, and deemed positive if a second band developed where the rK28 or rK39 had been coated. A test was deemed negative if only the control band developed.

Results were read in sufficient day light and assisted by a standard light source. Photographs were taken from each RDT within the respective allowed time for result reading interpretation. All RDTs were interpreted as negative or, if positive, the intensity of the test line was scored against the control line's intensity (1: weaker than; 2: equal to; 3: stronger than control line). DAT and RDTs were interpreted independently by two lab technicians who were blinded to the reference test results and clinical information. In case of discrepancies between the technicians, a third person and/or photographs were used as a tiebreaker.

DNA was extracted from Giemsa-stained spleen or bone marrow smear slides using the LEV DNA extraction kits (Promega, The Netherlands) with the Maxwell 16 automate (Promega) according to the manufacturer's instructions with a slightly adapted first step. Briefly, 30 µL of lysis buffer from the kit was dropped onto the smear and the material was scraped off with a sterile Bistouri knife and added to a DNA/RNA free 2 mL tube containing a final volume of 300 µL lysis buffer. After a quick spin, proteinase K was added, and the standard procedure was further followed. The kDNA PCR was performed as described before [15].

Data management and analysis

Data was recorded on register forms and entered in a Microsoft Excel database (Microsoft Corporation, Redmond, Washington, USA). Statistical analysis was done by use of Analyse-it for Excel (version 5.40.2). Test performance was analyzed by calculating sensitivity and specificity with Wilcoxon 95% confidence intervals for each test. Sensitivities between HIV positive and HIV negative patients were assessed for statistical significance using a two-tailed Fisher-exact test ($p<0.05$). Pearson Chi square (95% significance level) was used for correlation of results with parasite density. Inter-observer agreement was assessed by determining weighted kappa-values based on the scores given during RDT interpretation and interpreted according to Landis and Koch [16]: 1.00–0.81 excellent, 0.80–0.61 good, 0.60–0.41 moderate, 0.40–0.21 weak and 0.20–0.00 negligible agreement. Also, one weak positive sample was tested five times on different days to evaluate reproducibility.

Ethical review

Samples from VL suspect cases were leftovers from previous VL studies conducted at the LRTC. Amendments were made to the initial study protocols and approvals were obtained from the Institutional Review Board Antwerp (Belgium), Ethical Committee of the University Hospital Antwerp (Belgium) and the Institutional Review Board of the University of Gondar (Ethiopia) and the Ethiopian National Ethics Committee. According to ITM policy, approved by the Institutional Review Board, leftover samples of patients presenting at the ITM can be used for test evaluations as long as the patient's identity is not disclosed to third parties and the patient does not explicitly state an objection. For the endemic healthy controls informed consent was obtained prior to testing with approval of the Institutional Review Board of University of Gondar (Ethiopia).

Results

Test performance

No invalid test results were obtained for any of the tests. Performance of all evaluated tests in both HIV and non-HIV VL patients and controls are presented in Table 1. Overall, sensitivities ranged from 69.2% for DAT with cut-off ≥ 1/3200 to 92.2% for the Onsite RDT. Sensitivity was lower in the HIV positive group for all tests, though this was only statistically significant for rK39 Serion ELISA (p=0.0084) and OnSite RDT (p=0.0159) for which sensitivity in the non-HIV group reached 100%. DAT titers and rK39 Serion ELISA results according to HIV coinfection are illustrated in Fig 1. For DAT there was no significant difference (p= 0.1856) between titer proportions of the HIV positive and HIV negative group, opposed to rK39 Serion ELISA (p= 0.0270).

Table 1 Sensitivities and specificities of DAT, rk39 ELISA and six RDTs for VL diagnosis

	company	detection of	antigen	Sensitivity % (95% CIs) All (n=91)	HIV (n=51)	Non-HIV (n=40)	Specificity % (95% CIs) All (n=40)	number of false reactives EC (n=20)	NEC (n=10)	Malaria (n=10)
**DAT**										
≥800	ITM	Total antibodies	*Ldon promastigote*	**75.8 (66.1 - 83.5)**	70.6 (57.0 - 81.3)	82.5 (68.1 - 91.3)	**NA**	NA	NA	NA
≥3200				**69.2 (59.1 - 77.8)**	64.7 (51.0 - 76.4)	75.0 (59.8 - 85.8)				
**ELISA IgG**										
rK39 IgG	Serion	IgG antibodies	*Leish spp* rK39	**91.2 (83.6 - 95.5)**	84.3 (72.0 - 91.8)	100.0 (91.2-100.0)	**97.5 (87.1 - 99.6)**	0	0	1*
**RDTs**										
IT Leish	BioRad	IgG antibodies	rK39	**91.2 (83.6 - 95.5)**	88.2 (76.6 - 94.5)	95.0 (83.5 - 98.6)	**100 (91.2 - 100)**	0	0	0
Kalazar Detect	InBios	IgG antibodies	rK39	**89.0 (80.9 - 93.9)**	84.3 (72.0 - 91.8)	95.0 (83.5 - 98.6)	**92.5 (80.1 - 97.4)**	0	1	2&
rK39 IgG1	Coris	IgG1 antibodies	rK39	**76.9 (67.3 - 84.4)**	74.5 (61.1 - 84.5)	80.0 (65.2 - 89.5)	**100 (91.2 - 100)**	0	0	0
rK28 IgG1	Coris	IgG1 antibodies	rK28	**75.8 (66.1 - 83.5)**	70.6 (57.0 - 81.3)	82.5 (68.1 - 91.3)	**97.5 (87.1 - 99.6)**	0	0	1$
Kalazar Antigen ICT	Xinjier	antigen	NA	**89.0 (80.9 - 93.9)**	88.2 (76.6 - 94.5)	90.0 (76.9 - 96.0)	**20.0 (8.1 - 41.6)**	ND	8	8!
OnSite	CTK	Total antibodies	rK28	**92.2 (84.8 - 96.2)**	86.0 (73.8 - 93.0)	100 (91.2 - 100)	**77.5 (62.5 - 87.7)**	0	1	8µ

NA: not applicable, ND: not done; EC: endemic healthy controls, NEC: non-endemic controls
*1 *P. vivax* infection
& 1 *P. falciparum* and 1 *P. malariae* infection
$ 1 *P. vivax* infection
! 2 *P. falciparum*, 3 *P. vivax*, 1 *P. ovale*, 1 *P. malariae* and 1 *P. falciparum* and *P. ovale* mixed infection
µ 3 *P. falciparum*, 3 *P. vivax*, 1 *P. malariae* and 1 *P. falciparum* and *P. ovale* mixed infection

Fig 1: DAT and rK39 ELISA results stratified by VL-HIV coinfection (a) DAT titer distribution and (b) rK39 ELISA Serion U/mL results compared between VL-HIV co-infected (dark grey) and non-HIV VL patients (light grey).

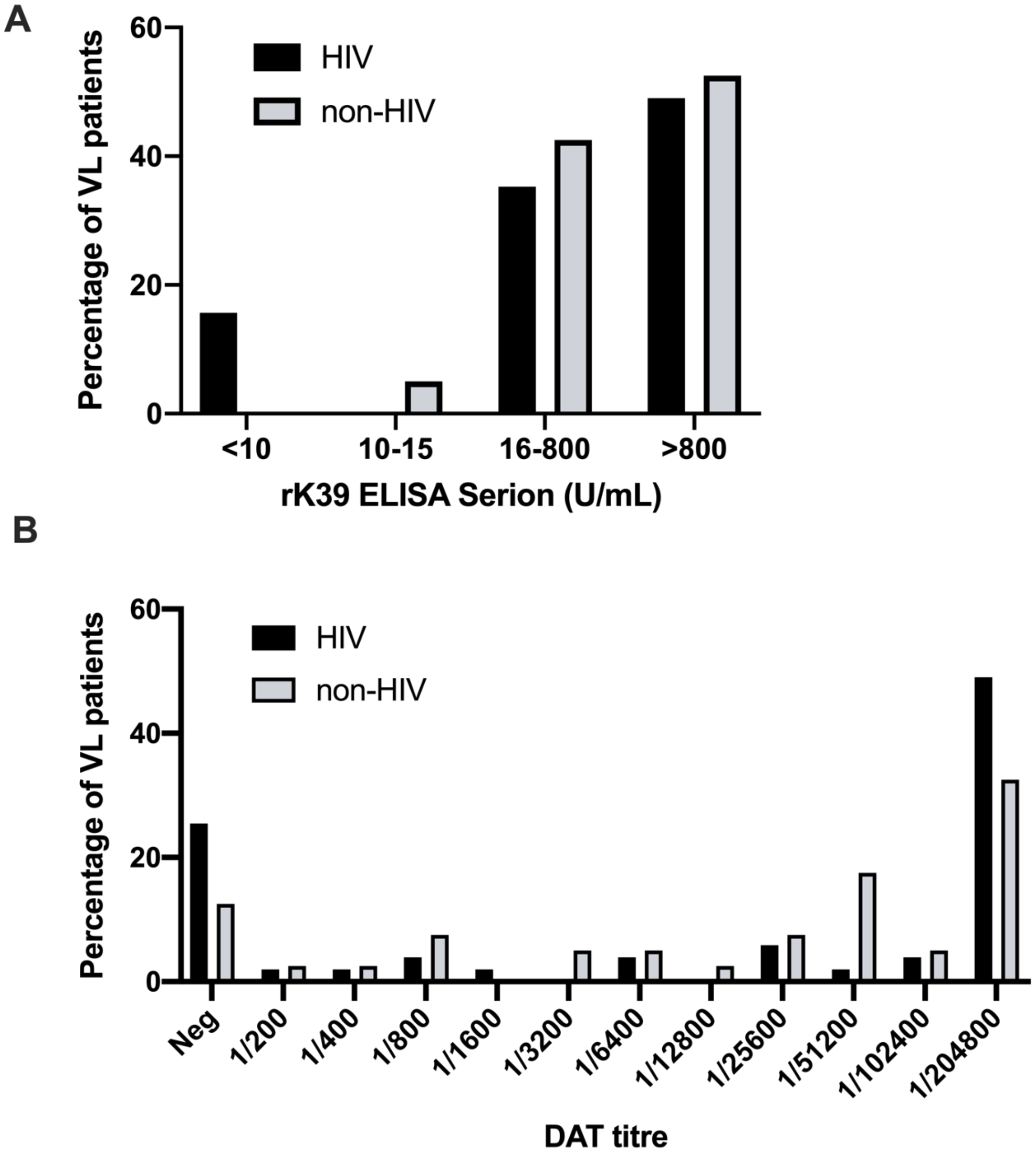

There was no statistically significant correlation between the test result proportions and parasite density for any of the tests except for DAT (Pearson Chi square p-value 0,0419 with cut-off ≥ 1/800 and 0.0019 with cut-off ≥ 1/3200). Also at a cut-off ≥ 1/3200, DAT was positive in 55 out of 74 (74.3%) microscopy positive cases, and only in 8 out of 17 (47.1%) microscopically negative cases (confirmed through PCR on slide).

Overall specificities ranged from 20,0% for Kalazar ICT to 100.0% for IT Leish, and rK39 IgG1. None of the tests were positive in the 20 endemic healthy controls. All 10 non-endemic control samples tested negative by all tests, except for 1 sample that was positive with the Kalazar Detect RDT, 1 other sample for Onsite RDT and 8 samples with the Kalazar ICT. One malaria positive sample tested positive with both rK28 IgG1 and Leishmania IgG ELISA, though still within borderline limits. Two other samples were found positive with the Kalazar Detect RDT and eight with Kalazar ICT and Onsite RDT.

Agreement between two tests was calculated (Table 2). The highest agreement was noted between rk39 Serion ELISA and IT Leish (96.9%) and the lowest between DAT at cut-off ≥ 1/3200 and Kalazar ICT (56.8%). Median values obtained by rK39 ELISA were compared across qualitative results.

Table 2. Percentage of concordant results between all tests

% concordant (n=131)	rK39 ELISA	IT Leish	Kalazar Detect	rK39 IgG1	rK28 IgG1	Kalazar Ag ICT	OnSite
DAT ≥ 1/800	87.8	87.8	84.7	84.0	81.7	62.2	85.4
DAT ≥ 1/3200	84	84.7	82.4	82.4	79.4	56.8	75.4
rK39 ELISA Serion		96.9	95.4	90.1	87.8	69.4	96.2
IT Leish			92.4	90.1	87.8	71.2	94.6
Kalazar Detect				90.1	84.7	71.2	91.5
rK39 IgG1					91.6	63.1	87.7
rK28 IgG1						62.2	86.9
Kalazar Antigen ICT							71.8

Inter observer agreement and reproducibility

Overall % agreement between readers in positive and negative test results was 100% for all tests, except for rK39 IgG1 (99.2%) and rK28 IgG1 (97.5%). The number of samples for which both readers differed in intensity reading was 3/131 (2.3%) for Onsite RDT and 3/111 (2.7%) Kalazar ICT, 6/131 (4.6%) for Kalazar Detect, 7/131 (5.3%) for IT-Leish, 16/131 (12.2%) for rK28 IgG1 and 18/131 (13.7%) for rK39 IgG1. Weighted kappa values for between-reader line-intensity readings were calculated for each RDT and all were equal or above 0.89. Test results were reproducible with only one discordance in line intensity over five days for rK39 IgG1 remaining within one category of difference (rated 1 to 2).

Discussion

With this study we aimed to assess diagnostic accuracy of multiple tests for VL diagnosis in HIV-coinfected Ethiopian patients as data are scarce. Our data have shown that sensitivity decreased in the HIV-VL coinfected group with some tests being more affected than others. To date, there is a lack of evidence for the minimum sensitivity and specificity of a single test or algorithm for VL diagnosis. As clinical presentation lacks specificity, and treatment is toxic, laboratory confirmation is necessary. Previously it was recommended to have a minimum sensitivity of 95% [17] and the test should be able to make a distinction between asymptomatic patients, treated patients and acute diseased patients. Current Ethiopian national guidelines on primary or *de novo* VL diagnosis start with a non-invasive anti-rK39 RDT upon presentation of clinical symptoms [18]. If positive, the patient should be considered as a new VL case and receive treatment. If negative, a DAT test is performed with a positive cut-off titer of ≥ 1/3200 and negative ≤1/400. DAT positive patients are referred for treatment, borderline results should

be repeated or splenic, bone marrow, lymph node aspiration could be considered if safe. Rarely a DAT negative result would require aspiration. There is no different algorithm for HIV patients, and even with reduced immune response due to HIV infection, sensitivity is still considered acceptable in the national guideline. An algorithm with high sensitivity to rule out VL infection and a high specificity to avoid these invasive sampling procedures and unnecessary treatment is needed.

Overall, none of the tests evaluated here reach an individual sensitivity of 95% and sufficiently high specificity. When applying the current algorithm to these results with the most sensitive RDT (Onsite RDT), DAT and sequential aspiration, still 6 out of 90 (6.7%) cases (all HIV positive patients) went undetected and 95% sensitivity was not reached either in our study. As per previous study protocol, 5/6 of these patients underwent splenic or bone marrow aspiration and obtained an aspirate positive (grades 2 to 6) result but they may not have been tested according to the algorithm because of a negative DAT result. Replacing DAT by the second most sensitive RDT (IT-Leish), would result in detection of one additional case. Remarkably, DAT was less sensitive in comparison with previous reported East African results, ranging from 83.5% to 99.1% [11,19-26]. Of note, sensitivities of DAT are often compared against a parasitological diagnosis obtained through microscopy which already lacks sensitivity. This was also shown here as 17 additional cases were afterwards detected through PCR on slides reported negative by microscopy. Assays detecting total antibodies against rK39 all showed a comparable sensitivity ranging from 89.0% to 91.2%. There was no significant difference in this study with sensitivity from the Onsite RDT detecting antibodies against rK28, though previously this has been reported to be higher in East African patients compared to rK39 antigen based RDTs [10-11]. Presumably the reduced sensitivity of rK39 RDTs in East Africa may be a reflection of molecular diversity of the kinesin gene [27], or by human genetic differences moreover as VL is mainly a pediatric disease in the Indian subcontinent compared to East Africa where all age

groups are affected [11]. Not only throughout continents, but also differences in sensitivities within a country may occur, as recently reported by a group from Brazil [28]. Albeit the Onsite RDT exhibits a high sensitivity, a problem implementing this as a screening test is its low specificity in a malaria endemic setting, as 8 out of 10 acute malaria cases reacted false positive (Table 1). Malaria is highly endemic in Ethiopia with an estimated of almost 3 million new cases in 2016 [29]. Prevalence of VL/malaria co-infection in Ethiopia remains largely unknown, but in the Metema hospital in Ethiopia a prevalence of 4.3% has been reported [30]. Aside from coated antigens, other elements influence performance, e.g. the format (ELISA, RDT, lateral-flow vs dipstick…), conjugate used, concentrations of components, sample type (serum, plasma or whole blood) and sample volume applied, etc. The Onsite RDT requires a higher sample volume for testing compared to the other RDTs. Dilution of sera has previously been successful in eliminating cross-reactivity against malaria [24], however this may negatively impact sensitivity. An rK39 antigen used in RDT format showed lower sensitivity in Sudan compared to Indian patients, but the same antigen in an ELISA format, results in comparable sensitivities across countries [24]. Here, sensitivity for the anti-rK39 ELISA was comparable to the anti-rk39 RDTs detecting IgG antibodies. None of the RDTs intrinsic quality suffered from invalid results, inter-reader variability or reproducibility problems.

Though these RDTs are implemented in diagnostic settings, they do not distinguish between an acute and treated infection as they detect either IgG or total antibodies. The only antigen detecting test evaluated here did not prove valuable because of its very low specificity. Previously, IgG1 was found to be the predominant antibody present in VL infection and has been explored as a potential biomarker of post-chemotherapeutic relapse [31-32]. Therefore it was hypothesized that IgG1 detection may also be useful in predicting progression to VL infection among asymptomatic patients which was previously supported by some limited data [32]. Rates of asymptomatic *Leishmania* infection in endemic regions in Ethiopia are estimated

to be 10-20% [33] and there is a need for biomarkers to predict progression. Data on the IgG1 titers and sensitivity in VL diagnosis and especially HIV/VL coinfected patients are still sparse [32]. Here, the 2 prototype IgG1 detection antibody tests against rK39 and rK28 antigens showed lower sensitivities compared to total IgG antibody detection when used as a diagnostic assay. Sensitivity in Indian VL patients for the rK39 IgG1 RDT was previously reported to be 94,7% - 100% depending on the concentration of antigen used [34]. The lower sensitivity obtained here could be due to different population characteristics between continents. However, samples in the study by Mollett *et al* were selected based on a rK39 total IgG positive test and microscopy at diagnosis, which likely explains the higher sensitivity they found. The observed results confirms that the IgG1 RDT's are not intended for use as a VL diagnostic assay but as a test of cure or relapse. Moreover these assays prototypes sensitivities may be improved by increasing reagent concentrations, combining rK28 and rK39 antigens or using *Leishmania* lysate as previously described [32].

In northwest Ethiopia, up to 40% of VL cases are HIV co-infected [12] and this is likely an underestimation as only 17% of VL cases are screened for HIV [35]. Current guidelines recommend HIV screening in VL cases [36]. However, HIV screening should already be performed upon clinical suspicion of VL to guide correct diagnostic algorithms. Sensitivities from all assays decreased upon stratification by HIV status, but the impact of an HIV co-infection was significant for two tests, the rK39 Serion ELISA and the Onsite RDT. Lower sensitivities are a corroboration of previous results [20,28] and likely due to the functional impairment of cell-mediated immunity and thus lower antibody responses. Gradoni *et al* [37] suggested that the sequence in which both infections are acquired may impact the seropositivity for VL, unfortunately it is often impossible to retrieve which infection was acquired first. Antibody titers in the rK39 ELISA were significantly lower in the HIV coinfected group, though this was not significant for DAT. In contrast to our results, a meta-analysis proved DAT to be the most

effective serological technique in the immunosuppressed by HIV infection [20]. Some tests were 100% sensitive in the non-HIV infected group and could be used to rule out VL infection. However, the current algorithm does not allow to rule out VL among the HIV infected. Agreement of test results between assays were calculated to assess if other algorithms would be of interest or tests could be used in parallel to increase sensitivity and specificity, however with the tests evaluated here this is not possible. Even when combining all antibody detection tests together, still four out of 51 HIV VL coinfected cases would go undiagnosed. Current antibody test cannot reliably rule out VL infection in HIV infected patients. Recently, a test was developed combining the advantages of both DAT and RDTs, with a leishmanial membrane extract used in an RDT format [38]. In Ethiopia this test had 100% sensitivity, but low specificity. It could be a valuable tool to rule out VL in the future, but more data are needed, especially in HIV patients. Other antigens have been explored, like rKLO8 and rKE16 [24,33], but also demonstrated regional differences in performance.

Limitations of this study are (i) it's relatively small sample size, (ii) samples were analyzed retrospectively and might have undergone multiple freeze-thaw cycles, with a negative impact on sensitivity, however, antibodies are unlikely to decrease substantially and this would apply to all assays evaluated, (ii) results from patients in control groups were compared against DAT as a reference test as opposed to PCR and/or microscopy which may introduce verification bias, (iii) PCR was not systematically performed on all slides; however, microscopy positive results are highly specific and less sensitive compared to PCR and this would unlikely have changed the results, (iii) we have no information on what infection was acquired first, HIV or VL, nor on HIV disease status or HAART, which may influence antibody titers.

Performance of commercial antibody detecting RDTs was variable and sub-optimal. Improvement of sensitivity and specificity with better diagnostic tests is needed. The IgG1-based assays are not appropriate as screening assays due to low sensitivity, but need to be

assessed as tools for follow-up of treatment. Assay performance depends on regional differences, associated with parasite divergency and on patient characteristics. Therefore, it is strongly recommended to select the appropriate tests and algorithm to a specific endemic setting and verify performance before implementing it. Separate algorithms for HIV coinfected against non-HIV infected would be valuable. Negative serology does not reliably rule out VL infection in HIV patients. This highlights the need to establish clear guidelines on minimal performance criteria of individual tests and algorithms, but also against which reference these should be compared as parasitological identification through microscopy only has low sensitivity. Large prospective evaluations of both serological and molecular tests taking all these variables into account are required to enable creation of clear guidelines for use of tests in the field.

Acknowledgments

We want to acknowledge all staff from the Leishmania Research and Treatment Centre, Gondar, Ethiopia for their cooperation on this project. We thank Michael Miles and Tapan Bhattacharya for providing the rK28 antigen for development of the IgG1 rapid test.

References

1. Boelaert, M., Verdonck, K., Menten, J., Sunyoto, T., van Griensven, J., Chappuis, F., et al. (2014). Rapid tests for the diagnosis of visceral leishmaniasis in patients with suspected disease. *The Cochrane database of systematic reviews*, *2014*(6), CD009135. https://doi.org/10.1002/14651858.CD009135.pub2

2. Global Health Observatory Data Repository. Number of cases of visceral leishmaniasis reported: Data by country (last update 05/02/2020; consulted on 13/04/2020). https://apps.who.int/gho/data/node.main.NTDLEISHVNUMIM?lang=en

3. Minakaran N, Soorma T, Ladhani SN. Visceral Leishmaniasis in a UK Toddler following a Short Trip to a Popular Holiday Destination in Spain. Case Rep Infect Dis. 2014; 537052. doi:10.1155/2014/537052

4. Reithinger R, Dujardin JC. Molecular diagnosis of leishmaniasis: current status and future applications. J Clin Microbiol. 2007; 45(1):21-25. doi:10.1128/JCM.02029-06

5. Peeling RW, Holmes KK, Mabey D, Ronald A. Rapid tests for sexually transmitted infections (STIs): the way forward. Sex Transm Infect. 2006; 82 Suppl 5:v1-v6. doi:10.1136/sti.2006.024265

6. Sundar S, Rai M. Laboratory diagnosis of visceral leishmaniasis. Clin Diagn Lab Immunol. 2002; 9(5):951-958. doi:10.1128/cdli.9.5.951-958.2002

7. Zijlstra E, Ali MS, El-Hassan A, El-Toum IA, Satti M, Ghalib H, et al. Kala-azar: a comparative study of parasitological methods and the direct agglutination test in diagnosis. Trans R Soc Trop Med Hyg. 1992; 86(5):505-7.

8. Chakravarty J, Hasker E, Kansal S, Singh OP, Malaviya P, Singh AK, et al. Determinants for progression from asymptomatic infection to symptomatic visceral leishmaniasis: A cohort study. PLoS Negl Trop Dis 2019; 13(3): e0007216. https://doi.org/10.1371/journal. pntd.0007216

9. World Health Organization. Visceral leishmaniasis rapid diagnostic test performance, Diagnostics Evaluation Series no.4, ISBN 978 92 4 150223 8

10. Pattabhi S, Whittle J, Mohamath R, El-Safi S, Moulton GG, et al. Design, Development and Evaluation of rK28-Based Point-of-Care Tests for Improving Rapid Diagnosis of Visceral Leishmaniasis. PLoS Negl Trop Dis 2010; 4(9): e822. doi:10.1371/journal.pntd.0000822

11. Bezuneh, A, Mukthar M, Abdoun A, Teferi T, Takele Y, Diro E et al. Comparison of Point-of-Care Tests for the Rapid Diagnosis of Visceral Leishmaniasis in East African Patients. Am J Trop Med Hyg. 2014; 91. 10.4269/ajtmh.13-0759.

12. Diro E, Lynen L, Ritmeijer K, Boelaert M, Hailu A, et al. Visceral Leishmaniasis and HIV Coinfection in East Africa. PLOS Negl Trop Dis 2014; 8(6): e2869. doi:10.1371/journal.pntd.0002869

13. Bossuyt PM, Reitsma JB, The STARD initiative, The Lancet. 2003; 361, 9351 71, https://doi.org/10.1016/S0140-6736(03)12122-8.

14. World Health Organization. Control of the Leishmaniases. Geneva, Switzerland: WHO Technical Report Series 949, World Health Organization; 2010. Report of a meeting of the WHO Expert Committee on the Control of Leishmaniases, Geneva, March 22–26, 2010.

15. van Griensven J, van Henten S, Mengesha B, Kassa M, Adem E, Endris Seid M,

et al. Longitudinal evaluation of asymptomatic Leishmania infection in HIV-infected individuals in North-West Ethiopia: A pilot study. PLoS Negl Trop Dis. 2019;13(10):e0007765

16. Landis JR and Koch GG, Biometrics, Vol. 33, No. 1 (1977), pp. 159-174. DOI: 10.2307/2529310

17. Chappuis, F., Sundar, S., Hailu, A. et al. Visceral leishmaniasis: what are the needs for diagnosis, treatment and control? Nat Rev Microbiol 2007; 5, 873–882 https://doi.org/10.1038/nrmicro1748

18. Federal Ministry of Health of Ethiopia. Guideline for diagnosis, Treatment and Prevention of Leishmaniasis in Ethiopia: Second edition June 2013

19. ter Horst R, Tefera T, Assefa G, Ebrahim AZ, Davidson RN, Ritmeijer K. Field evaluation of rK39 test and direct agglutination test for diagnosis of visceral leishmaniasis in a population with high prevalence of human immunodeficiency virus in Ethiopia. Am J Trop Med Hyg. 2009 Jun;80(6):929-34.

20. Cota GF, de Sousa MR, Demarqui FN, Rabello A. The diagnostic accuracy of serologic and molecular methods for detecting visceral leishmaniasis in HIV infected patients: meta-analysis. PLoS Negl Trop Dis. 2012;6(5):e1665. doi:10.1371/journal.pntd.0001665

21. Cota G, de Sousa MR, Mara de Freitas Nogueira B, Gomes LI, Oliveira E, Santana et al.

Comparison of Parasitological, Serological, and Molecular Tests for Visceral Leishmaniasis in HIV-Infected Patients: A Cross-Sectional Delayed-Type Study. Am J Trop Med Hyg, 2013; 89,3 pp 570 – 577 DOI: https://doi.org/10.4269/ajtmh.13-0239

22. Maia Z, Lírio M, Mistro S, Mendes CM, Mehta SR, Badaro R. Comparative study of rK39 Leishmania antigen for serodiagnosis of visceral leishmaniasis: systematic review with meta-analysis. PLoS Negl Trop Dis. 2012;6(1):e1484. doi:10.1371/journal.pntd.0001484

23. Cañavate C, Herrero M, Nieto J, Cruz I, Chicharro C, Aparicio P, et al. Evaluation of Two rK39 Dipstick Tests, Direct Agglutination Test, and Indirect Fluorescent Antibody Test for Diagnosis of Visceral Leishmaniasis in a New Epidemic Site in Highland Ethiopia. Am J Trop Med Hyg, 2011; 84;1 pp102 – 106. https://doi.org/10.4269/ajtmh.2011.10-0229

24. Abass E, Kang C, Martinkovic F, Semião-Santos SJ, Sundar S, Walden P, et al. Heterogeneity of Leishmania donovani Parasites Complicates Diagnosis of Visceral Leishmaniasis: Comparison of Different Serological Tests in Three Endemic Regions. PLoS ONE 2015; 10(3): e0116408. doi:10.1371/journal.pone.0116408

25. Mukhtar M, Abdoun A, Ahmed AE, Ghalib H, Reed SG, Boelaert M, et al. Diagnostic accuracy of rK28-based immunochromatographic rapid diagnostic tests for visceral leishmaniasis: a prospective clinical cohort study in Sudan. Trans R Soc Trop Med Hyg, 2015; 109, 9, pp594–600, https://doi.org/10.1093/trstmh/trv060

26. Ayelign B, Jemal M, Negash M et al. Validation of in-house liquid direct agglutination test antigen: the potential diagnostic test in visceral Leishmianiasis endemic areas of

Northwest Ethiopia. BMC Microbiol 2020: 20, 90. https://doi.org/10.1186/s12866-020-01780-0

27. Bhattacharyya T, Boelaert M, Miles MA. Comparison of Visceral Leishmaniasis Diagnostic Antigens in African and Asian Leishmania donovani Reveals Extensive Diversity and Region-specific Polymorphisms. PLoS Negl Trop Dis 2013 7(2): e2057. doi:10.1371/journal.pntd.0002057

28. Sanchez MCA, Celeste BJ, Lindoso JAL, Fujimori M, de Almeida RP, Fortaleza CMCB, et al. Performance of rK39-based immunochromatographic rapid diagnostic test for serodiagnosis of visceral leishmaniasis using whole blood, serum and oral fluid. PLoS ONE 2020: 15(4): e0230610. https://doi.org/10.1371/journal.pone.0230610

29. Tadele G, Teha S, Misgun S. Burden of malaria in Ethiopia, 2000-2016: findings from the Global Health Estimates 2016.Trop Dis Travel Med Vaccines. 2019; 5: 11.

30. Ferede G, Diro E, Getie S, Getnet G, Takele Y, et al. Visceral Leishmaniasis-malaria co-infection and their associated factors in patients attending Metema Hospital, Northwest Ethiopia. Suggestion for integrated vector management. Malar Res Treat. 2017 https://doi.org/10.1155/2017/6816913

31. Bhattacharyya T, Ayandeh A, Falconar AK, Sundar S, El-Safi S, et al. IgG1 as a Potential Biomarker of Post-chemotherapeutic Relapse in Visceral Leishmaniasis, and Adaptation to a Rapid Diagnostic Test. PLoS Negl Trop Dis 2014; 8(10): e3273. doi:10.1371/journal.pntd.0003273

32. Marlais T, Bhattacharyya T, Singh OP, Mertens P, Gilleman Q, Thunissen C, et al. Visceral Leishmaniasis IgG1 Rapid Monitoring of Cure vs. Relapse, and Potential for Diagnosis of Post Kala-Azar Dermal Leishmaniasis. Front. Cell. Infect. Microbiol. 2018; 8:427. doi: 10.3389/fcimb.2018.00427

33. Abass E, Bollig N, Reinhard K, et al. rKLO8, a novel Leishmania donovani - derived recombinant immunodominant protein for sensitive detection of visceral leishmaniasis in Sudan. PLoS Negl Trop Dis. 2013;7(7):e2322. Published 2013 Jul 18. doi:10.1371/journal.pntd.0002322

34. Mollett G, Hinckel BCB, Bhattacharyya T, Marlais T, Singh OP, Mertens P, et al. Detection of Immunoglobulin G1 Against rK39 Improves Monitoring of Treatment Outcomes in Visceral Leishmaniasis. Clin Infect Dis. 2019; 69, 7, pp1130–1135, https://doi.org/10.1093/cid/ciy1062

35. WHO (2010) Leishmaniasis. www.who.int/leishmaniasis/resources/ETHIOPIA.pdf (accessed June 6th 2020)

36. van Griensven J, Ritmeijer K, Lynen L, Diro E. Visceral leishmaniasis as an AIDS defining condition: towards consistency across WHO guidelines. PLoS Negl Trop Dis 2014; 8: e2916

37. Gradoni L, Scalone A, Gramiccia M. HIV-Leishmania co-infections in Italy: serological data as an indication of the sequence of acquisition of the two infections. Trans R Soc Trop Med Hyg 1993; 87, 1, pp 94–96, https://doi.org/10.1016/0035-9203(93)90441-R

38. Ejazi, SA, Ghosh S, Saha S et al. A multicentric evaluation of dipstick test for serodiagnosis of visceral leishmaniasis in India, Nepal, Sri Lanka, Brazil, Ethiopia and Spain. Sci Rep 2019; 9, 9932. https://doi.org/10.1038/s41598-019-46283-9
