# Supplementary material for: Diagnostic accuracy of direct agglutination test, rK39 ELISA and six rapid diagnostic tests among visceral leishmaniasis patients with and without HIV coinfection in Ethiopia
Source: PLoS Negl Trop Dis. 2020 Dec 31;14(12):e0008963. doi: 10.1371/journal.pntd.0008963 (PMC7774845; doi:10.1371/journal.pntd.0008963)
Supplement: Attachment — Submitted filename: Diagnostic accuracy of direct agglutination test.doc [file pntd.0008963.s002.doc]

**Diagnostic accuracy of direct agglutination test, rK39 ELISA and six rapid diagnostic tests among visceral leishmaniasis patients with and without HIV coinfection in Ethiopia**

PNTD-D-20-01173

**General comments:**

1) Studies aimed at evaluation of diagnostic tests, many a time, are seen to show a lot of variability even when the same tests are used in different conditions and settings. As a result, recommendations made are also varied. The source of variability is multiple. There is widespread problem in reproducibility of test results between even between different brands of the same test. Some products come from different companies and in different formats. Protocols and study designs for performance evaluation of diagnostic tests are also varied, and sample sizes are calculated in many different ways. In this study, there is no sample size calculation, and the sample size is considered small (page 18; line 351). What is the implication of this in the validity of the results and the recommendations?

2) One wonders how endemic countries will absorb such varied results of diagnostic studies and the recommendations made. It is to be noted that endemic countries have developed their guidelines based upon the results of earlier studies.

I would like to know the opinion of the authors which of the results among the so far published works can be considered reliable. This is indirectly asking for unambiguous recommendation concerning diagnostics for VL.

3) The title of the manuscript refers to diagnostic accuracy. In line with this, I was expecting to see DOR (Diagnostic Odds Ratio) or Diagnostic effectiveness/ accuracy, and even ROC curves; at least one of these. If such metrics cannot be provided, then, the title may better begin as “A study on diagnostic performance of DAT, rk39 ……. In the diagnosis of VL patients with and….”.

**Specific comments:**

1. What does the expression “de novo” mean in the context of the manuscript? It is used several times.

2. Previously, DAT has been considered as a reference test since many field studies were carried out in different endemic regions, and also PCR tests were not fully optimized in the past. In fact, its sensitivity had been around 92% and above. So, it is surprising that the data in this manuscript is showing much lower sensitivity even in HIV negative patients.

3. Page 5 (line 88), there is a missing word in the phrase “commercially available differs…”

4. Page 5 (line 97) seems to miss a phrase or a word in the section that reads as “………. Indian subcontinent but has…..”

5. Page 5 (line 102) has a phrase “within and between” in the sentence that describes the objective of the study. The phrase “within and between” probably suggest the comparative nature of the study. But this is not implied or stated in the same sentence.

6. In page 6 (line 111), the STARD guideline is mentioned, but not presented. There is no STARD diagram.

7. In page 6 (line 118), there appears to be a missing word before the word “density’ in the phrase “stratified by density and HIV status”. The missing word is likely to be “parasite”.

8. In page 6 (lines 126-128), it is mentioned that endemic control specimens were subjected to screening by DAT before testing by the other tests. Why was DAT used for screening? Did the authors believe that it was sensitive enough to rule out Leishmania infection? Why was PCR not used given that it was used as a reference test in this study? The pre-screening selects the “good” negatives and hence, the specificity of the tests will be overestimated. This raises a question of validity of the results related to specificity. For evaluation of diagnostics using stored samples, it would have been better not to include samples from endemic regions.

9. Figure 1 legends “a” and “b” are interchanged.

10. In page 13, the sentences between lines 233 and 239 are not easy to comprehend and appear contradictory. Particularly, the second sentence is confusing. It first states that all the 10 non-endemic controls were negative by all tests, but then mentions numbers positive by some of the tests.

It is also mentioned that none of the tests were positive in samples of endemic controls. Obviously this should be expected knowing that the samples were pre-screened.

The last sentence of the paragraph is also unclear what it is referring to. It mentions numbers of samples positive in other samples. What are these “other samples”?

It is better to re-write the paragraph.

11. In page 14 (lines 248 & 249), there is data about percent agreement between readers. I am surprised that there is no variability at all. At least in DAT, we expect to see some variability as DAT titres are based on determination of end points. Often there is a difference of 1 or 2 titres.

In Page 14 (line 266), the phrase “primary de novo VL” is written. What does de novo mean here?

12. In page 15 (line 273), the statement “An algorithm with high sensitivity to rule out VL…..” is given. The expression “rule out” does not go with “high sensitivity”. Perhaps rule-in a better expression.

13. In page 15 (lines 276 & 277), the end of the sentence is wrongly constructed. Split into two sentences or correct accordingly.

14. In page 15 (line 278), the phrase “sequential aspiration” is mentioned, but lacks clarity as the notion was not mentioned in the materials and methods section.

15. In page 25 (line 280), the phrase “5/6 of these patients” can be written as “5 of these 6 patients”

16. In page 17 (line 330), the phrase “Moreover these assays prototypes sensitivities…. “ is confusing.

17. In page 17 (lines 339/340), the statement “likely due to the functional impairment of cell mediated immunity and thus lower antibody responses”, is trying to explain the causes of lower sensitivity of the tests in HIV co-infected VL patients. The statement lacks support by reference citation, and the cell mediated immunity is too broadly presented. It might be better to refer to impaired Th2 responses.

18. The terms “rule out VL” are used several times. What does it connote? Can it be replaced by “to diagnose VL”?

19. In page 18 (lines 361/362), there is mention of results from patients in control groups being compared against DAT as a reference test as opposed to PCR and/or microscopy. This was stated differently in the materials and methods section. In fact, the endemic controls were pre-screened, not compared. It is also considered as a limitation, but it would better qualify as shortfall.

20. The lack of information about which infection (HIV or VL) was acquired first (page 18; lines 365/366) is indeed a relevant issue, but it makes little sense to consider it a limitation of the study. The study could not address this issue anyway.
