# Supplementary material for: Diagnostic accuracy of direct agglutination test, rK39 ELISA and six rapid diagnostic tests among visceral leishmaniasis patients with and without HIV coinfection in Ethiopia
Source: PLoS Negl Trop Dis. 2020 Dec 31;14(12):e0008963. doi: 10.1371/journal.pntd.0008963 (PMC7774845; doi:10.1371/journal.pntd.0008963)
Supplement: Attachment — Submitted filename: Reviewers comments response_20201007.doc [file pntd.0008963.s004.doc]

**Response to reviewers - Diagnostic accuracy of direct agglutination test, rK39 ELISA and six rapid diagnostic tests among visceral leishmaniasis patients with and without HIV coinfection in Ethiopia**

PNTD-D-20-01173

We would like to thank the editor and reviewers for their nice words and constructive comments. In what is to follow we provide our answers to each of the comments given and indicate how we have incorporated them into the revised version of the manuscript. Line numbers are provided according to the tracked changes document.

Reviewer 1:

Major comments:

1. The investigators noted (page 5, line 98) that VL-HIV coinfection rate in Ethiopia range from 20 to 40%, citing a reference that is relatively old. Nonetheless, a more recent systematic review and meta-analysis demonstrated that overall prevalence of HIV in VL ranges from 20.9% to 24.9% (Mohebali M and Yimam Y. BMC Infect Dis 2020; 20:214. doi:10.1186/s12879-020-4935-x). Thus, this statement needs revisiting. In addition same should be done discussion part (line 333).

*At the time of writing we were not yet aware of this interesting recent publication. We thank the reviewer for pointing this out. We adapted the references and the manuscript text in lines 100 - 102 and line 343 accordingly.*

2. Methods section (page 6, line 128): What is the DAT result of the ten Belgian travelers (Non-endemic controls)? The reviewer assumes that the results are negative. If so, the authors need to explicitly mention it.

*These samples were all negative, this is now added in line 138.*

3. Methods section: In page 7, line 141, the authors used two different cut-off titers in interpreting DAT results, namely ≥ 1/800 and ≥ 1/3200, given that DAT is considered as negative if titer is ≤ 1/400, positive at ≥ 1/3200, and borderline at ≥ 1/800. No justification was provided, however, for using such two different cut-offs. Needs explanation. In addition, where do we put/classify those DAT titers with ≥ 1/1600?

*In the package insert of DAT a negative result is ≤ 1/400, borderline from 1/800 to 1/3200 and positive ≥ 1/6400, when the end-dilutions are being considered. Sensitivity and specificity varies according to the cut-off chosen and in literature different cut-offs can be chosen depending on the (endemic) region. In the Ethiopian National guidelines a negative result is considered* ≤ 1/400, borderline 1/800 to 1/1600 and positive ≥ 1/3200 *Here we selected these two cut-offs (at borderline and positive interpretation) as this also impacts further actions to obtain a diagnosis as outlined in the discussion (line 270-272). Titers of 1/1600 would be considered positive in case of cut-off* ≥ 1/800 and negative in case of cut-off ≥ 1/3200. *The reason why these cut-offs were chosen is now added in the text in lines 147-148.*

4. What are the reasons why borderline rK39-ELISA were considered as positive in the analyses (line 147)? Why not as negative?

*This is often debated in terms of interpreting performance of serological tests. Here we did not work with two different cut-offs (as compared to DAT), because this ELISA test is not well described in literature (as opposed to DAT) and the fact that there is no established guideline on what to do with borderline results (as opposed to DAT). We chose to consider such results as positive and provide this information explicitly to the reader. It is of note that the number of rK39 ELISA borderline results are few, as shown in Fig 1A. The number of borderline results (n=3) is now added to Table 1 in line 225 for clarification.*

5. Line 171: DNA was extracted from Giemsa-stained tissue smear slides. Is there any data known previously the accuracy of the results in using DNA from Giemsa-stained smears? Will this impact on the results as PCR was used in this study as a ‘reference test’ for assessing the sensitivity of the different assays for VL diagnosis employed in this study.

*There are only limited data with regards to Leishmania DNA extracted from Giemsa stained smears for both cutaneous and visceral leishmaniasis. Brustoloni et al demonstrated that PCR showed significant higher sensitivity than microscopy and 16 out of 19 cases with negative microscopy tested positive by PCR on slides. Pandey et al were able to additionally identify VL in 12% of samples which were negative by microscopy. Unfortunately we did not have the samples to make a comparison between PCR on tissue aspirates with PCR performed on smears from these aspirates. Also, the aspirates were not kept and we only had the smears available. The aim of using this PCR on smears was merely to be able to confirm suspect VL cases as true VL cases in addition to only microscopy to select true positive cases for the sensitivity panel. The performance of the reference test indeed influences the sensitivity of the comparator tests. Therefore, it is important when comparing results across studies that the reference test is taken into account upon interpretation. It was highlighted in the conclusion that guidelines are needed to specify which reference test should be used (line 388).*

6. Line 212: With respect to Figure 1 on titer proportions of the HIV positive vs. HIV negative VL group – which DAT titer proportions are you discussing? At ≥ 1/800 or ≥ 1/3200? or both? Needs clarifications. In addition, you need to present exact data on proportions (with 95% CI) of rK39-ELISA (line 213) so that the reader can see the differences easily. Having p value only is not suffice.

*The aim was to see whether there were differences in proportions of reported DAT titers or rK39 ELISA result groups existed between the HIV negative and the HIV positive population. The meaning of proportions was clarified by adding ‘number of patients with a given titer or ratio’ in line 219. E.g. if there are 20 HIV positive patients and 15 HIV negative patients with a DAT titer of 1/800, 12 HIV positives and 15 HIV negatives with titer 1/3200, etc, it is verified whether these proportions are statistically equal across titers or not. It is unclear what is meant by exact data with 95%CI, as this is not possible in our opinion.*

7. The lower sensitivity (≈77%, all cases) of rK39 IgG1 RDT in Ethiopian VL (line 325) was unexpected when compared with the 94.7% to 100% sensitivity among Indian VL cases. Several reasons were provided by the investigators. One notable difference is the fact that in the Indian study, the ‘reference assay’ used was parasitology only, whereas for the Ethiopian study, it was either parasitology or PCR. Have you tried to dis-aggregated data analysis by ‘reference test’ (74 VL cases by microscopy vs 17 VL cases by PCR), and whether the discrepancies in the sensitivities between the two countries persisted?

*We did calculate this separately. The proportions of IgG1 positives are shown below. When disaggregating data the sensitivity in the microscopy confirmed group does increase but not substantially up to the levels seen in the Indian setting. Several reasons were named for this geographical difference. The text was slightly adapted on line 338.*

|  | **Reference: microscopy positive (n=74)** | **Reference: PCR positive (n=17)** |
| --- | --- | --- |
| **rK39 IgG1** | 59/74 (79,7%) | 11/17 (74,7%) |
| **rK28 IgG1** | 57/74 (77,0%) | 12/17 (70,6%) |

Minor comments:

-	Please check for proper citations of the references (e.g. reference #s: 11, 19, 21, 23 etc…..)

*It is unclear what is meant by the reviewer. The citations mentioned were checked and these correspond to the correct references. All references were checked and corrected to correspond to the correct format. (attention: in the revised version reference numbers have changed because of addition of a new reference).*

**Reviewer 2:**

**General comments:**

1) Studies aimed at evaluation of diagnostic tests, many a time, are seen to show a lot of variability even when the same tests are used in different conditions and settings. As a result, recommendations made are also varied. The source of variability is multiple. There is widespread problem in reproducibility of test results between even between different brands of the same test. Some products come from different companies and in different formats. Protocols and study designs for performance evaluation of diagnostic tests are also varied, and sample sizes are calculated in many different ways. In this study, there is no sample size calculation, and the sample size is considered small (page 18; line 351). What is the implication of this in the validity of the results and the recommendations?

*We did not perform sample size calculation as this was a retrospective study with a sample set based on the availability and usability (according to ethical approvals) of samples. When a sample size is small, this influences the confidence intervals of the results which were added in Table 1 for clarity. In addition, some clarification was added in line 369. The aim of this study was to compare the performance of different tests for the diagnosis of VL and not to approve/disapprove a single test as fit for diagnosis. Larger effects can already be picked up in a smaller sample size and provide valuable information (e.g. malaria cross-reactivity for one of the tests, limited sensitivity for all tests). Also evaluation of new prototypes, even on small sample sizes to start with, is useful to see if a one test diagnosis could be possible (which was not the case here for IgG1 detection as it has a limited sensitivity for diagnosing VL). These results could guide in further selection of a test for proper extended validation for screening/diagnostic purposes. It is impossible to derive the proper algorithm based solely on these results. And even a meta-analysis making a clear conclusion from different publications is difficult, as different standards are being used. That is why in the conclusion it is discussed that adequate guidelines should be developed with desirable criteria for performance and even still than local evaluation to a specific setting is needed. We feel that this was sufficiently addressed in the manuscript and it would not change our recommendations.*

2) One wonders how endemic countries will absorb such varied results of diagnostic studies and the recommendations made. It is to be noted that endemic countries have developed their guidelines based upon the results of earlier studies.

I would like to know the opinion of the authors which of the results among the so far published works can be considered reliable. This is indirectly asking for unambiguous recommendation concerning diagnostics for VL.

*This was addressed in the discussion and, please, also see the response to the previous question. We feel that endemic countries should not solely rely on what is published in earlier studies. Some publications refer to the improved sensitivity of rK28 over rK39 in East Africa, however this was not significant in our study and there even was a specificity issue due to malaria infection. Based on this (limited) information one cannot start introducing rK28 tests in algorithms. Algorithms should be developed and evaluated thoroughly before implementing them. Also regular revision is needed as tests improve and golden standards may change. Currently it is not possible to give a clear recommendation as these large scale studies are missing. For the same reason, it is neither possible to confirm that the current Ethiopian algorithm is still the best one. Based on current knowledge and performance of the available tests, the Ethiopian algorithm can be used. However tests like DAT and rK39 were evaluated against microscopy or a consensus diagnosis (based on symptoms) and not PCR which has improved sensitivity. When large studies and/or assays with improved performance become available, re-evaluation of the algorithm would be valuable.*

*It is not always feasible for researchers or a single institute to do this on a large scale but governments could invest in establishing national reference centers who are given the means and mandate to perform such studies. Large organizations (eg FIND, WHO) also attempt to perform these diagnostic test evaluation studies, however for VL they are currently lacking.*

3) The title of the manuscript refers to diagnostic accuracy. In line with this, I was expecting to see DOR (Diagnostic Odds Ratio) or Diagnostic effectiveness/ accuracy, and even ROC curves; at least one of these. If such metrics cannot be provided, then, the title may better begin as “A study on diagnostic performance of DAT, rk39 ……. In the diagnosis of VL patients with and….”.

*Different measures of diagnostic accuracy exist, like the ones mentioned by the reviewer. Sensitivity/specificity is also a measure of diagnostic accuracy, which is available in the manuscript. Thus the title of the manuscript is not misleading in that way. However, if the editor agrees that the title should be changed, we suggest the following:*

*‘Comparison of DAT, rK39 ELISA and six rapid diagnostic tests for the diagnosis of visceral leishmaniasis in Ethiopia’*

**Specific comments:**

1. What does the expression “de novo” mean in the context of the manuscript? It is used several times.

*De novo was changed throughout the manuscript in ‘first-time episode’ for clarification.*

2. Previously, DAT has been considered as a reference test since many field studies were carried out in different endemic regions, and also PCR tests were not fully optimized in the past. In fact, its sensitivity had been around 92% and above. So, it is surprising that the data in this manuscript is showing much lower sensitivity even in HIV negative patients.

*We agree and we were surprised as well that it was this low. That is why we hypothesized in line 293 that this may be related to the reference used, as is also proposed by the reviewer.*

3. Page 5 (line 88), there is a missing word in the phrase “commercially available differs…”

*The word ‘assays’ was added in line 90*.

4. Page 5 (line 97) seems to miss a phrase or a word in the section that reads as “………. Indian subcontinent but has…..”

*This phrase was adapted for clarification in line 99.*

5. Page 5 (line 102) has a phrase “within and between” in the sentence that describes the objective of the study. The phrase “within and between” probably suggest the comparative nature of the study. But this is not implied or stated in the same sentence.

*The aim of comparing tests was added to this sentence in line 106.*

6. In page 6 (line 111), the STARD guideline is mentioned, but not presented. There is no STARD diagram.

*A STARD flow diagram was provided in the new Supplementary Figure 1 and referred to in line 116.*

7. In page 6 (line 118), there appears to be a missing word before the word “density’ in the phrase “stratified by density and HIV status”. The missing word is likely to be “parasite”.

*This was added in line 122.*

8. In page 6 (lines 126-128), it is mentioned that endemic control specimens were subjected to screening by DAT before testing by the other tests. Why was DAT used for screening? Did the authors believe that it was sensitive enough to rule out Leishmania infection? Why was PCR not used given that it was used as a reference test in this study? The pre-screening selects the “good” negatives and hence, the specificity of the tests will be overestimated. This raises a question of validity of the results related to specificity. For evaluation of diagnostics using stored samples, it would have been better not to include samples from endemic regions.

*We apologise for the confusion caused by this sentence and clarified it in line 132-138. For the endemic controls, healthy individuals were tested for all tests, and not preselected based on DAT results. We think it is valuable to include endemic healthy controls, as this may already show a specificity problem for a certain test.*

9. Figure 1 legends “a” and “b” are interchanged.

*This was corrected in the figure legend.*

10. In page 13, the sentences between lines 233 and 239 are not easy to comprehend and appear contradictory. Particularly, the second sentence is confusing. It first states that all the 10 non-endemic controls were negative by all tests, but then mentions numbers positive by some of the tests.

It is also mentioned that none of the tests were positive in samples of endemic controls. Obviously this should be expected knowing that the samples were pre-screened.

The last sentence of the paragraph is also unclear what it is referring to. It mentions numbers of samples positive in other samples. What are these “other samples”?

It is better to re-write the paragraph.

*This paragraph has been adapted for clarification in line 241 – 247.*

*As mentioned in response to question 8, the healthy endemic controls were not pre-screened. DAT was used as a reference standard and all included controls, tested negative. None were excluded because of a positive DAT result.*

11. In page 14 (lines 248 & 249), there is data about percent agreement between readers. I am surprised that there is no variability at all. At least in DAT, we expect to see some variability as DAT titres are based on determination of end points. Often there is a difference of 1 or 2 titres.

*In line 193, in the materials and methods section, it is mentioned that these calculations are performed for RDTs. This was also clarified in line 257. We did not do this for DAT as this is already a well-known phenomenon.*

In Page 14 (line 266), the phrase “primary de novo VL” is written. What does de novo mean here?

*This was changed to first-time episode.*

12. In page 15 (line 273), the statement “An algorithm with high sensitivity to rule out VL…..” is given. The expression “rule out” does not go with “high sensitivity”. Perhaps rule-in a better expression.

*When a test has a high sensitivity and the pretest probability is high (e.g. in a patient suspected for VL based on the clinical definition), then a negative result means that VL can be safely ruled out, since a very sensitive test would unlikely miss a diagnosis. Therefore, we did not change the sentence, and hope the reviewer can agree.*

13. In page 15 (lines 276 & 277), the end of the sentence is wrongly constructed. Split into two sentences or correct accordingly.

*This was corrected in line 282.*

14. In page 15 (line 278), the phrase “sequential aspiration” is mentioned, but lacks clarity as the notion was not mentioned in the materials and methods section.

*The sentence starts with ‘when applying the current algorithm…’. This algorithm is explained in the paragraph before. We did not actively follow or evaluate the algorithm in this study and thus this was not mentioned in the materials and methods section. We explained this phrase better by replacing it by ‘aspiration if required’ in line 286.*

15. In page 25 (line 280), the phrase “5/6 of these patients” can be written as “5 of these 6 patients”

*This was adapted in line 287.*

16. In page 17 (line 330), the phrase “Moreover these assays prototypes sensitivities…. “ is confusing.

*Some words were removed in line 340 for simplification.*

17. In page 17 (lines 339/340), the statement “likely due to the functional impairment of cell mediated immunity and thus lower antibody responses”, is trying to explain the causes of lower sensitivity of the tests in HIV co-infected VL patients. The statement lacks support by reference citation, and the cell mediated immunity is too broadly presented. It might be better to refer to impaired Th2 responses.

*This was specified in line 349 and a reference was added.*

18. The terms “rule out VL” are used several times. What does it connote? Can it be replaced by “to diagnose VL”?

*It means ‘to exclude’ VL. This was added in line 282, at first use, so it is clear further on in the text what is meant by it.*

18. In page 18 (lines 361/362), there is mention of results from patients in control groups being compared against DAT as a reference test as opposed to PCR and/or microscopy. This was stated differently in the materials and methods section. In fact, the endemic controls were pre-screened, not compared. It is also considered as a limitation, but it would better qualify as shortfall.

*It is impossible to compare results from control groups to PCR and/or microscopy as it is unethical to collect tissue aspirates, which is invasive and includes risks, in these individuals. However, to have a reference for comparison across these controls, we used DAT. Ofcourse, this entails that, given the lower sensitivity, it may possibly be that another test is positive and it could be a true positive, however, given the low pre-test probability, this is rather unlikely. We consider this a limitation, rather than a shortfall and have adapted the sentence in line 374 for clarification why it was done this way.*

19. The lack of information about which infection (HIV or VL) was acquired first (page 18; lines 365/366) is indeed a relevant issue, but it makes little sense to consider it a limitation of the study. The study could not address this issue anyway.

*It is correct that because of the retrospective approach that this could not be done, however, even when collecting data prospectively in HIV and VL time of infection, this remains difficult as both infections can go unnoticed for a long time and it is hard to retrieve date of infection. We wanted to draw the attention that this is relevant when interpreting results. When new evaluation studies would be performed, it is something that can be included through inquiring the patient or based on previous laboratory results, if available. Though there is also referral to this in the discussion in line 352, we prefer to keep it within the limitations paragraph as well. However if the editor disagrees, we can remove it.*

Reviewer 3:

Abstract: It is a little confusing; at the beginning of the abstract, the number of tests need to identify the number of RDTs evaluated in the study. In the lower section of the abstract, the reader doesn't know exactly what tests the authors are referring to as compared with the initial sentence. The abstract has to be self explanatory, not assuming that the reader will read the whole article.

*We assume the reviewer refers to the ‘six RDTs’ and that these should be specified already in the abstract. These were added in line 26-28.*

The last sentence used in the abstract and conclusion, is odd and needs to be rephrased "Clear guidelines on minimal performance criteria of individual tests and algorithms, but also against which reference these should be compared are needed."

*This sentence was rephrased in line 43.*

The term de novo is not commonly used and it could be replaced or explained.

The medical dictionary states:

de novo

1. Over again from the beginning; anew.

2. Previously undetected.

3. Previously untreated.

*De novo was changed throughout the manuscript in ‘first-time episode’ for clarification.*

Line 118, correct this sentence "...no previous VL history and stratified by PARASITE density and HIV status"

*The word ‘parasite’ was added to this sentence*

How were the DAT cutoff dilutions established?

*This was based on Ethiopian guidelines, and added in line 147 in the materials and methods section.*

Table 1. Why was "NA" used in % specificity, etc. for DAT?

*DAT was used as the reference test for control samples. Therefore, it could not be calculated. This was more clearly explained in line 138 and was already reported in the limitations of the study.*

Figure 1. The order of graphs was inverted in the legend

*This was corrected in the figure legend.*
